# Redundancy and compensation in axon guidance: genetic analysis of the *Drosophila *Ptp10D/Ptp4E receptor tyrosine phosphatase subfamily

**DOI:** 10.1186/1749-8104-3-3

**Published:** 2008-01-31

**Authors:** Mili Jeon, Huong Nguyen, Sami Bahri, Kai Zinn

**Affiliations:** 1 Broad Center, Division of Biology, California Institute of Technology Pasadena, California 91125, USA; 2 Institute of Molecular and Cell Biology, 61 Biopolis Drive, Proteos 138673, Singapore

## Abstract

**Background:**

*Drosophila *has six receptor protein tyrosine phosphatases (RPTPs), five of which are expressed primarily in neurons. Mutations in all five affect axon guidance, either alone or in combination. Highly penetrant central nervous system (CNS) and motor axon guidance alterations are usually observed only when specific combinations of two or more RPTPs are removed. Here, we examine the sixth RPTP, Ptp4E, which is broadly expressed.

**Results:**

Ptp4E and Ptp10D are closely related type III RPTPs. Non-drosophilid insect species have only one type III RPTP, which is closest to Ptp10D. We found that *Ptp4E *mutants are viable and fertile. We then examined *Ptp4E Ptp10D *double mutants. These die before the larval stage, and have a mild CNS phenotype in which the outer longitudinal 1D4 bundle is frayed. *Ptp10D Ptp69D *double mutants have a strong CNS phenotype in which 1D4 axons abnormally cross the midline and the outer and middle longitudinal bundles are fused to the inner bundle. To examine if *Ptp4E *also exhibits synthetic phenotypes in combination with *Ptp69D*, we made *Ptp4E Ptp69D *double mutants and *Ptp4E Ptp10D Ptp69D *triple mutants. No phenotype was observed in the double mutant. The triple mutant phenotype differs from the *Ptp10D Ptp69D *phenotype in two ways. First, the longitudinal tracts appear more normal than in the double mutant; two or three bundles are observed, although they are disorganized and fused. Second, axons labelled by the SemaIIB-τMyc marker often cross in the wrong commissure. We also examined motor axon guidance, and found that no phenotypes are observed in any *Ptp4E *double mutant combination. However, triple mutants in which *Ptp4E Ptp10D *was combined with *Ptp69D *or *Ptp52F *exhibited stronger phenotypes than the corresponding *Ptp10D *double mutants.

**Conclusion:**

Type III RPTPs are required for viability in *Drosophila*, since *Ptp4E Ptp10D *double mutants die before the larval stage. Unlike Ptp10D, Ptp4E appears to be a relatively minor player in the control of axon guidance. Strong phenotypes are only observed in triple mutants in which both type III RPTPs are eliminated together with Ptp69D or Ptp52F. Our results allow us to construct a complete genetic interaction matrix for all six of the RPTPs.

## Background

Signalling via tyrosine phosphorylation is essential for axon guidance in many systems. Target proteins involved in signal transduction and cytoskeletal dynamics in growth cones are phosphorylated by tyrosine kinases (TKs) and dephosphorylated by tyrosine phosphatases (PTPs).

In a simplified view of phosphotyrosine pathways controlling cell growth and differentiation, signaling is triggered by engagement of receptor tyrosine kinases (RTKs) by ligands. Ligand binding induces receptor dimerization and phosphorylation of downstream targets. RTK signalling is downregulated by dephosphorylation of autophosphorylated RTKs and other signalling molecules by cytoplasmic PTPs. In this scenario, the PTPs are passive modulators of a process in which the 'informational' event that initiates signalling is ligand binding to the RTK.

In contrast, phosphotyrosine signalling pathways involved in growth cone guidance in the *Drosophila *embryonic central nervous system (CNS) involve receptor tyrosine phosphatases (RPTPs) and cytoplasmic TKs. Like RTKs, RPTPs are modular signalling receptors. They have cell adhesion molecule-like extracellular (XC) domains, linked via a single transmembrane region to one or two cytoplasmic PTP domains. Five of the six fly *Rptp *genes are selectively expressed in CNS neurons, and all of these genes have loss-of-function phenotypes that affect axon guidance [1-6].

The TK that is central to many growth cone guidance events in the *Drosophila *embryo is Abl, a cytoplasmic kinase [7-9]. *Drosophila *has many RTKs, but no functional RTK has been implicated in embryonic axon guidance (the kinase-related axon guidance receptors Derailed and Off-track are thought to lack enzymatic activity) [10,11]. These facts suggest that phosphotyrosine signalling in growth cones could be controlled in a manner opposite to that used in RTK pathways. In this scheme, the growth cone would use a cytoplasmic TK to constitutively phosphorylate targets, and the 'information' that alters signalling strength would be transmitted via engagement of RPTPs by ligands located on the surfaces over which the growth cone travels.

Of course, this is a greatly oversimplified picture, because there are many other receptors that can influence phosphotyrosine signalling in embryonic growth cones. For example, the Roundabout 1 (Robo1) receptor is an essential regulator of axon guidance across the midline. Phosphorylation of Robo1 by Abl may be regulated by Robo1's engagement of its ligand Slit, and in this case the 'information' that triggers signalling would be delivered via Slit binding to Robo1 [9]. Also, it is unlikely that phosphorylation by Abl is an unregulated, constitutive process. Nevertheless, it is striking that the receptors are kinases and the cytoplasmic modulators are phosphatases in pathways that regulate cell growth, while the reverse seems to be true for pathways that control neuronal growth cone guidance.

RPTP pathways are poorly understood relative to RTK pathways, partially because *in vivo *ligands that regulate axon guidance and synaptogenesis have been identified only for the *Drosophila *Lar RPTP. These are the heparan sulfate proteoglycans Syndecan and Dallylike [12,13]. However, Lar also has non-heparan sulfate proteoglycan ligands [14,15], and ligands for the other five fly RPTPs have not yet been defined. It has also been difficult to identify substrates that are important for RPTP signalling *in vivo*.

Five *Drosophila *RPTPs have been genetically characterized in published papers. Four of these (Ptp10D, Lar, Ptp69D, Ptp99A) are expressed only on CNS axons in late embryos [16-18], and the fifth, Ptp52F, is CNS-specific but is expressed on both axons and cell bodies [5]. All of the published zygotic phenotypes for these genes are alterations in axon guidance, suggesting that this is the major function of this gene family in *Drosophila*. In contrast, many mammalian RPTPs are expressed in non-neural tissues and have functions unrelated to axon guidance.

The RPTPs regulate both CNS and motor axon guidance. There is extensive redundancy among the five genes, so that highly penetrant guidance phenotypes are usually observed only when two or more RPTPs are genetically removed. Studies of motor axon guidance indicate that each guidance decision made by motoneuron growth cones requires a specific subset of the RPTPs. For example, axons in the ISNb nerve are unable to defasciculate from the common ISN pathway in *Lar Ptp69D Ptp99A *triple mutants. The later decision by ISNb axons to enter their target muscle field fails in *Lar *single mutants, so that the axons bypass the muscle field, but the bypass phenotype is suppressed and muscle field entry restored in *Lar Ptp99A *double mutants [19]. This example illustrates that RPTPs can exhibit either functional redundancy, in which the absence of one RPTP is compensated for by another RPTP, or competition, in which removal of a second RPTP suppresses the guidance errors caused by the absence of the first RPTP. Similar genetic interactions among RPTPs may also occur in vertebrates, as two recent papers show that double mutant combinations and RNA interference perturbations involving the vertebrate Lar and type III (Ptp10D-like) RPTP subfamilies produce complex alterations in motor axon guidance [20,21].

In this paper, we examine the functions of the sixth and last *Drosophila *RPTP, Ptp4E. This protein is closely related to Ptp10D, and is the product of a recent gene duplication. Unlike the other RPTPs, *Ptp4E *is widely expressed in late embryos. When we began these studies, we thought that *Ptp4E *mutations might have phenotypes affecting many non-neural tissues, since loss of Ptp4E could not be compensated for by neural-specific RPTPs. However, our findings show that *Ptp4E *single mutants have no detectable phenotypes, because Ptp4E is redundant with the closely related Ptp10D. Double mutant embryos lacking both of these RPTPs die at hatching, but they have specific phenotypes affecting only CNS axons and tracheal cells.

Here we describe the axon guidance phenotypes produced by *Ptp4E *mutant combinations. The tracheal phenotypes will be described elsewhere (MJ and KZ, manuscript in preparation). The data in this paper, together with those in earlier papers from our group [4,5,19], allow us to construct complete pairwise interaction matrices that define how all six *Drosophila *RPTPs regulate CNS and motor axon guidance.

## Results and discussion

### Evolution of *Ptp4E*

**Figure 1:**
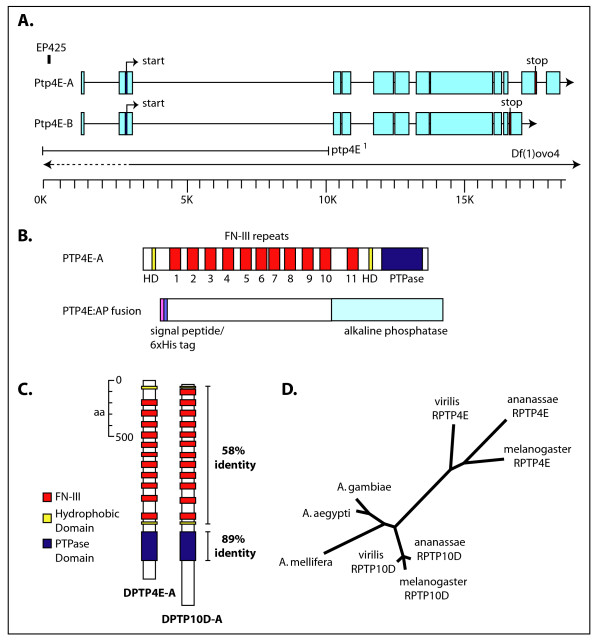
Organization of the *Ptp4E *gene and protein, and evolutionary analysis. **(a) **The EP425 element that was used to generate the *ptp4E*^1 ^allele was inserted 1,157 nucleotides upstream of the transcription start site. The portions of the gene deleted in *ptp4E*^1 ^and *Df(1)ovo4 *are shown with a solid line when confirmed by PCR, and with a dotted line to show that the endpoint lies within the dotted region. *Df(1)ovo4 *is the smallest deficiency that removes *Ptp4E*. **(b) **Domain organization of Ptp4E protein, and the segment that was included in the 4E-AP construct. Ptp4E consists of a hydrophobic region (HD) and 11 fibronectin type III repeats (FN-III) in the extracellular (XC) domain, and a single putative catalytic phosphatase domain in the intracellular region. The 4E-AP construct that was used to express protein consists of a secretion signal peptide sequence (pink), 6× His residues (blue) followed by Ptp4E XC domain and human placental alkaline phosphatase sequence. **(c) **Comparison of Ptp4E ad Ptp10D protein structure arrangement and sequence comparison. Ptp4E and Ptp10D are very similar in structure and are 54% identical along the entire length of the protein. **(d) **Phylogenetic tree indicating that non-drosophilid species have only one copy of *Ptp4E/Ptp10D*, which is more similar to Ptp10D than to Ptp4E. Ptp10D is likely to be the ancestral copy. This is visualized in the tree by comparing the branch lengths connecting Ptp4E and Ptp10D to other insect species. *D. melanogaster *Ptp10D is closer than Ptp4E to the sequences in other insects that have only one type III RPTP gene, indicating greater sequence similarity. Also, the Ptp4E sequence evolved much faster than the Ptp10D sequence after the duplication (compare the branch lengths within the *melanogaster/ananassae/virilis *cluster for the two genes). *A. gambiae*, mosquito; *A. aegypti*, mosquito; *A. mellifera*, honey bee.

*Ptp10D *and *Ptp4E *are clearly the result of a gene duplication that occurred much more recently than the split between the other *Drosophila Rptp *genes. The amino acid sequences of their catalytic PTP domains share 89% identity, versus 36–40% identity for pairwise comparisons of *Ptp4E *with other *Drosophila *RPTPs. Their XC domains have a very similar organization, containing chains of 11 FN3 repeats in Ptp4E and 12 FN3 repeats in Ptp10D, and are 58% identical in amino acid sequence (Figure 1c). The *Ptp4E *gene encodes two predicted preproteins, of 1,767 and 1,607 amino acids, while the *Ptp10D *gene encodes preproteins of 1,931 and 1,631 amino acids. The sequences that differ between the alternative gene products are at the carboxyl terminus in both cases, but there is no sequence similarity between the Ptp4E and Ptp10D proteins within this region. Both genes reside on the X chromosome, and the nine *Ptp4E *introns within conserved coding sequence all correspond exactly in position to *Ptp10D *introns. *Ptp10D *has one additional intron not found in *Ptp4E*.

The *Caenorhabditis elegans *gene *dep-1 *is the ortholog of both *Ptp10D *and *Ptp4E*. Humans and mice have five genes encoding type III RPTPs, defined as proteins with XC domains composed of long chains of FN3 domains and a single PTP domain. Among these, the product of the PTPRB gene (PTPβ, not to be confused with RPTPβ, which is a different protein also known as PTPζ) has a somewhat higher alignment score to Ptp10D and Ptp4E than the other four mammalian type III proteins. These are: DEP-1/CD148, encoded by the *Ptprj *gene; PTPRO; SAP-1, encoded by the *Ptprh *gene; and PTPRQ. Since the radiation into the five mammalian genes seen today occurred after the split between arthropods and mammals, one cannot define any of the type III mammalian genes as an ortholog of one of the fly genes. A more complete description of the relationships among all the *Drosophila*, *C. elegans*, and mammalian RPTPs is found in [5].

The recent availability of genome sequences from twelve different *Drosophila *species, three mosquito species, two hymenopterans, a beetle, and the silkmoth allowed us to trace the evolution of the *Ptp10D/Ptp4E *gene pair within insect lineages. Surprisingly, we find that the *Ptp4E *gene is found only in drosophilid species. Mosquitoes, which are also dipterans, and all other sequenced insects have only a single *Rptp *gene corresponding to this gene pair. This gene is always much more closely related to *Ptp10D *than to *Ptp4E*. In addition, the *Ptp4E *sequence exhibits more sequence diversity among the drosophilid species than does the *Ptp10D *sequence. These data indicate that *Ptp10D *is the ancestral gene and that its sequence has been constrained more by evolution than the *Ptp4E *sequence since the time of the duplication. *Ptp4E *has evolved much more rapidly, suggesting that it may have acquired new function(s) since its emergence or was less essential for fitness than *Ptp10D*. These relationships are displayed in the phylogenetic tree of Figure 1d.

The *Ptp10D *ortholog found in all insect species always contains the *Ptp10D*-specific intron, and all *Ptp4E *orthologs in drosophilids lack this intron. This suggests that the intron may have been lost at the time of the duplication from the copy that evolved into *Ptp4E*. This would have been between 235 million years ago (the estimated time at which the mosquito and fly lineages diverged from each other) and 40 million years ago (the estimated time at which the radiation among the 12 sequenced drosophilid species occurred) [22,23].

We attempted to trace the history of the duplication by examining the genes adjacent to *Ptp10D *and *Ptp4E*, but found that the organizations of the *Ptp10D *and *Ptp4E *regions in *D. melanogaster *arose long after the *Ptp10D *and *Ptp4E *genes diverged from each other. *Ptp10D *is flanked by the *Rst(1)JH *and *bifocal *genes. *Rst(1)JH *is found upstream of the *Ptp10D *ortholog in both the *obscura *and *melanogaster *groups, but is separated from it in *D. willistoni *and all other drosophilids. *bifocal *orthologs are adjacent to the *Ptp10D *gene only in the *melanogaster *group. Similarly, the two genes flanking *Ptp4E*, *SIP3 *and *CG4068*, are located next to the *Ptp4E *ortholog only within the *obscura *and *melanogaster *groups. There are no significant sequence similarities between the genes that flank *Ptp10D *and *Ptp4E*. See [24] for a phylogenetic tree displaying the relationships among the sequenced insect species.

### Characterization of *Ptp4E *mutations

We generated a deletion mutation in *Ptp4E*, denoted *Ptp4E*^1^, by imprecise excision of a P element, EP425, located upstream of the putative transcription start site. This mutation removes the first and second exons, thus deleting the sequences encoding the initiating methionine and the first 67 amino acids of the Ptp4E protein (Figure 1a).

*Ptp4E*^1^*/Y *males are viable, fertile, and apparently healthy, as are *Ptp4E*^1^*/Df(1)ovo4 *females. We could not detect any alterations in the CNS or neuromuscular system in embryos or larvae of these genotypes. *Df(1)ovo4/Y *embryos also had no CNS or neuromuscular system phenotypes that could be detected by antibody staining. To determine if the absence of a loss-of-function phenotype for *Ptp4E *is due to compensation by the closely related Ptp10D protein, we constructed *Ptp4E*^1 ^*Ptp10D*^1 ^double mutants. *Ptp10D*^1 ^is an excision mutation that removes the amino-terminal coding sequences of *Ptp10D *[3]. *Ptp10D*^1 ^animals are viable and fertile, and also have no detectable defects in their embryonic nervous systems. In contrast, *Ptp4E*^1 ^*Ptp10D*^1^*/Y *animals can hatch out into first instar larvae, but die immediately after hatching. To ensure that this lethality is not due to other mutations on these chromosomes that confer lethality when combined in the *Ptp4E*^1 ^*Ptp10D*^1 ^recombinant chromosome, we also made a double mutant chromosome containing independently isolated insertions just upstream of *Ptp4E *(*Ptp4E*^*KG*2328^) and *Ptp10D *(*Ptp10D*^*EP*1172^). This chromosome is also lethal in hemizygous males (*Ptp4E*^*KG*2328 ^*Ptp10D*^*EP*1172^*/Y*). These data confirm the hypothesis that the viability of animals lacking either Ptp4E or Ptp10D is due to compensation by the other protein.

### Expression of *Ptp4E *mRNA and protein

The published *in situ *hybridization data suggest that *Ptp4E *mRNA is ubiquitously expressed in late embryos, although there are some level differences between tissues [25]. To further analyze expression, and to ensure that the observed pattern was not affected by cross-hybridization between the closely related *Ptp4E *and *Ptp10D *phosphatase domain sequences, we repeated this analysis using a probe from the first four *Ptp4E *exons, which are not closely related to *Ptp10D*.

**Figure 2:**
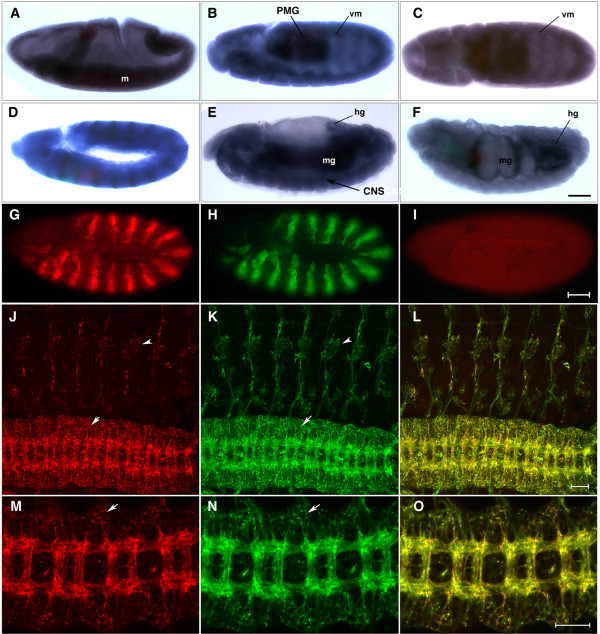
*Ptp4E *expression and protein localization during embryogenesis. **(a-f) **Expression of *Ptp4E *during development was visualized by *in situ *hybridization of whole-mount embryos. In all panels, anterior is left and dorsal is up, except for (c), which shows a ventral view. (a) At stage 8, in gastrulating embryos, *Ptp4E *expression is enriched in the mesoderm (labelled m). (b,c) In germ band extended stage 11 embryos, *Ptp4E *is expressed at highest levels in the posterior midgut primordium (PMG) and also shows a scalloped pattern that corresponds to the visceral mesoderm (vm). (d) As a positive control, we also stained embryos where UAS-Ptp4E-GFP was driven using the *engrailed*-GAL4 driver. The characteristic striped *engrailed *pattern is observed, indicating that the probe recognizes *Ptp4E *transcripts. (e) At stage 15, the strongest *Ptp4E *signal is in the midgut (mg); the ventral nerve cord (CNS) is also visible (arrow). (f) At stage 17, the gut is segmented and starts to coil. *Ptp4E *is expressed at highest levels at the anterior and posterior ends of the midgut and in the hindgut (hg). **(g-o) **Anti-Ptp4E antibodies recognize ectopically expressed Ptp4E-GFP. (g,h) The UAS-Ptp4E-GFP construct was ectopically expressed using the *engrailed-GAL4 *driver in stage 11 embryos. Anti-Ptp4E signal (g; red) colocalizes with theUAS-Ptp4E-GFP (visualized with anti-GFP; h, green) expression pattern, indicating that the antibody specifically recognizes the Ptp4E protein. (i) In wild-type embryos, the antibody shows a low and ubiquitous signal. Ptp4E-GFP protein was expressed in all postmitotic neurons using *elav*-GAL4 (j-o). Stage 16 embryo was visualized with anti-Ptp4E (j,m), anti-GFP (k,n), and merged (l,o). Ptp4E-GFP protein is transported out to the axons (m-o) as the CNS ladder brightly stains with anti-Ptp4E and anti-GFP. Ptp4E also accumulates in cell bodies, since the CNS region outside of the axon tracts stains brightly (j,k,m,n, arrows). PNS cell bodies (chordotonal organs) are also visible (j,k, arrowheads). (j-l) Images are projections of confocal stacks. (m-o) Images are single confocal sections. Scale bars are 35 microns (a-i) and 20 microns (j-o).

In gastrulating embryos, *Ptp4E *mRNA is enriched in the invaginating mesoderm (Figure 2a). In germ band extended embryos (stage 10–11), the strongest expression is observed in the posterior midgut primordium. There is also an interesting 'scalloped' pattern of expression observed at the ectodermal border (Figure 2b,c, vm). This has an intriguing correspondence to visceral mesoderm dpERK staining [26], suggesting that Ptp4E may be enriched at sites of RTK activation. Interestingly, *thisbe*, which encodes a ligand for the fibroblast growth factor receptor Heartless-, is expressed in a similar pattern [27]. Figure 2d is a germ-band extended embryo expressing a UAS-linked Ptp4E-green fluorescent protein (GFP) fusion from the *engrailed*-GAL4 driver. This shows the expected striped pattern of expression, confirming that the probe recognizes *Ptp4E *and allowing an estimate of the relative levels of driven versus endogenous *Ptp4E *mRNA.

At stage 14, *Ptp4E *is widely expressed, with highest levels observed in the midgut. Expression in the CNS can also be seen (Figure 2e). Finally, at stage 17 expression is much higher in the gut than elsewhere, with particularly strong expression observed in the hindgut and at the anterior end of the midgut (Figure 2f).

To examine Ptp4E protein expression and localization, we generated a variety of mouse monoclonal and polyclonal antibodies against Ptp4E. We made a construct that consisted of 6×-His-tagged Ptp4E XC domain fused to human placental alkaline phosphatase (PTP4E:AP; Figure 1b). PTP4E:AP was expressed in insect cells, purified over a Ni-NTA column, and injected into mice. To characterize the resulting antibodies, we first stained embryos that overexpressed Ptp4E-GFP using the *engrailed*-GAL4 driver. Figure 2g,h shows double staining of a germ-band extended embryo with anti-Ptp4E (red) and anti-GFP (green). The polyclonal antibodies clearly recognize the Ptp4E protein, as anti-Ptp4E (red) signal colocalizes with anti-GFP (green) signal in the expected striped pattern. Figure 2i shows a wild-type embryo at the same stage stained with anti-Ptp4E, where the expression appears to be ubiquitous. Note that the staining between the stripes in Figure 2g is much weaker than within the stripes, suggesting that the endogenous protein is expressed at low levels.

**Figure 3:**
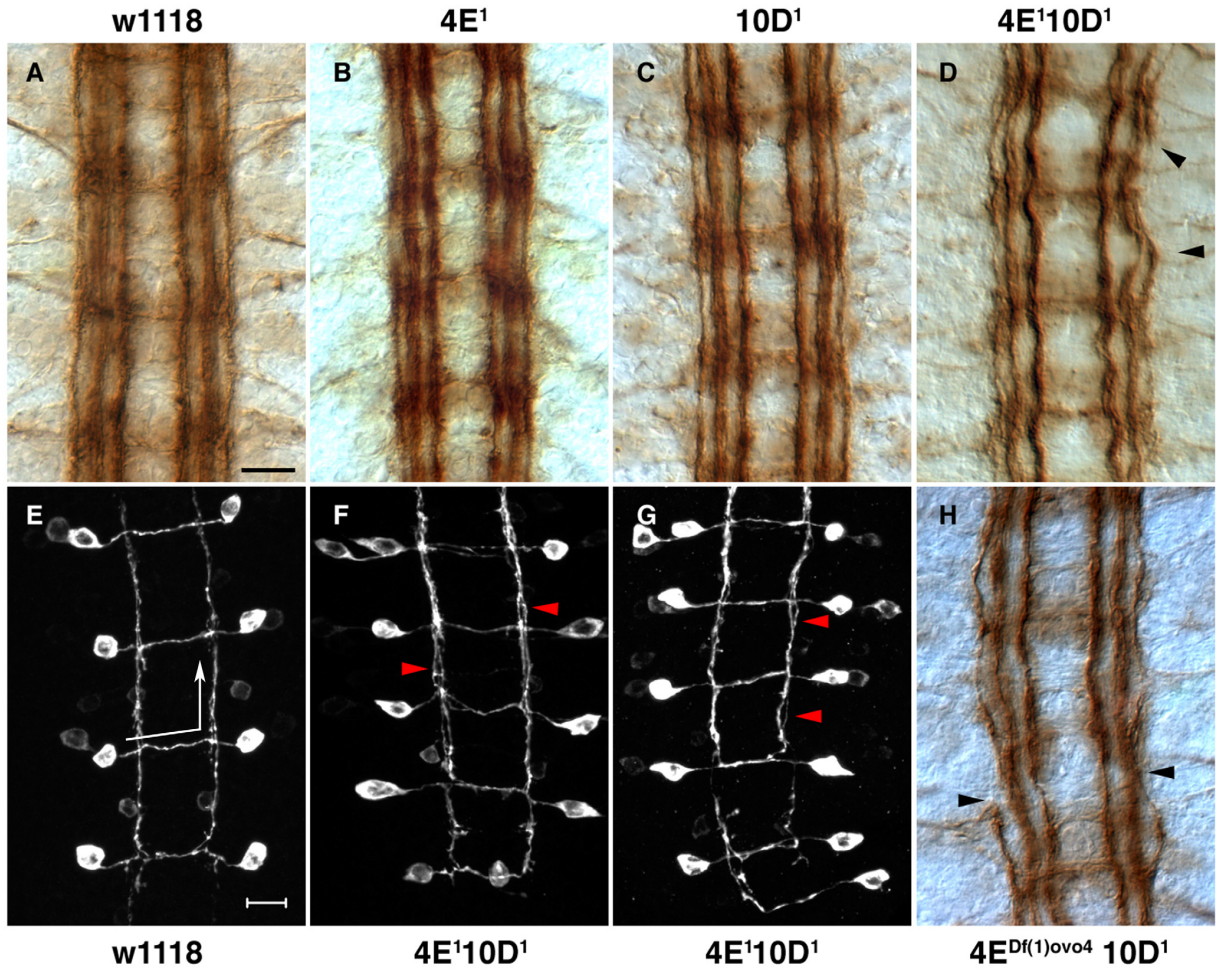
CNS phenotype of *Ptp4E Ptp10D *double mutants. **(a-d,h) **Stage 17 embryos were stained with mAb 1D4 and visualized using HRP immunohistochemistry. **(e-g) **Stage 17 embryos expressing the transgene SemaIIb-τmyc were stained with anti-Myc antibodies, and images are projections of confocal stacks. (a) Wild-type embryos at stage 17 show three distinct longitudinal tracts on either side of the midline. Commissural bundles do not stain with 1D4 at this stage, although cell bodies showing light staining are visible here. *Ptp4E*^1 ^(b) and *Ptp10D*^1 ^(c) embryos do not show any defects in CNS axon guidance, and are indistinguishable from control *w*^1118 ^CNS (a). The double mutants *Ptp4E*^1 ^*Ptp10D*^1 ^(d) and *Df(1)ovo4 ptp10D*^1 ^(h) show mild CNS defects. The longitudinal axons appear wavy and the outermost longitudinal axon tract is discontinuous and often invades the middle (intermediate) longitudinal axon tract (arrowheads). SemaIIB axons were visualized using the SemaIIb-τmyc transgene. (e) In the wild-type CNS, semaIIb-τmyc expressing axons project across the midline along the anterior commissural bundle and extend anteriorly along the intermediate 1D4 fascicle (arrows indicate the direction of axon trajectory). (f,g) SemaIIb-τmyc axons project in a normal manner in the double mutants. However, they have a consistent defect within their longitudinally projecting segment, in which the axon bundles do not form a tight bundle but have a frayed appearance (arrowheads). Anterior is up in all panels. Scale bars are 10 microns.

Although the ubiquitous staining observed with the antibody is consistent with the *in situ *hybridization data, we cannot be sure that antibody staining in wild-type embryos is due to Ptp4E protein, because it is not significantly reduced in *Ptp4E *mutant embryos (*Ptp4E*^1 ^or *Df(1)ovo4*). This finding could be explained in two ways. First, *Ptp4E*^1 ^mutants might continue to make an abnormal Ptp4E protein(s) due to initiation of translation at methionine residues encoded in exons not removed by the excision mutation (the second methionine residue in Ptp4E is at amino acid 377, within an undeleted exon). This protein, if it exists, would lack a signal sequence and may be nonfunctional, because the CNS phenotypes of *Ptp4E*^1 ^*Ptp10D*^1 ^and *Df(1)ovo4 Ptp10D*^1^*/Y *embryos are identical (Figure 3). *Df(1)ovo4 *deletes the entire *Ptp4E *gene (Figure 1). The presence of antibody staining in *Df(1)ovo4 *mutants could be due to persistence of Ptp4E protein synthesized from maternal mRNA, since early embryos contain large amounts of *Ptp4E *mRNA [25]. Second, it is possible that our Ptp4E antibodies cross-react with another ubiquitously expressed protein. They do not cross-react with Ptp10D, because the signal does not decrease in *Ptp10D*^1 ^mutant embryos.

Although we could not use the antibody to define where Ptp4E is expressed in the embryonic CNS in wild-type embryos, its ability to recognize overexpressed Ptp4E protein (Figure 2g,h) allowed us to ask whether Ptp4E can localize to axons. To do this, we drove Ptp4E-GFP with the pan-neuronal *elav*-GAL4 driver. In these embryos, bright staining of both CNS and peripheral nervous system (PNS) axons is observed with anti-Ptp4E and anti-GFP antibodies, and the two patterns are superimposable (Figure 2j–o). Interestingly, Ptp4E-GFP also appears to localize to neuronal cell bodies in the PNS and CNS (Figures 2j–l). In contrast, Ptp10D, Ptp69D, Lar, and Ptp99A, which are restricted to axons in wild-type embryos, are also axon-specific when overexpressed (unpublished data).

Expression of Ptp10D protein was detected only in the nervous system in published work. It is selectively expressed on embryonic CNS axons [16,17], and in the neuropil of the larval and adult brain [28]. Our recent data, however, show that Ptp10D is also expressed by embryonic tracheal cells. These findings suggest that the embryonic/larval lethality of *Ptp4E*^1 ^*Ptp10D*^1 ^animals might be due to either nervous system or tracheal phenotypes. In fact, we have found that these embryos have severe tracheal defects. These will be described elsewhere (MJ and KZ, manuscript in preparation). Their nervous system defects, however, are relatively mild (see below), and would not be expected to produce early lethality. Consistent with this, we find that GAL4-driven pan-neural expression of a UAS-Ptp4E-GFP fusion, which is capable of rescuing the tracheal phenotype when driven in tracheal cells by *breathless*-GAL4 (MJ and KZ, manuscript in preparation), does not rescue lethality in the *Ptp4E*^1 ^*Ptp10D*^1 ^background.

Driving Ptp10D in tracheae with *breathless*-GAL4 in a *Ptp4E*^1 ^*Ptp10D*^*EP*1172 ^background (the *EP1172 *line is a UAS-containing P element insertion upstream of the gene, so it allows rescue by crossing in GAL4 drivers) rescues lethality, allowing some adults to emerge. These data confirm that lethality in the double mutant is rescuable by Ptp10D expression in tracheae (or in other cells that express *breathless*-GAL4). We also attempted to rescue lethality by ubiquitous expression of Ptp4E, but found that pancellular overexpression of Ptp4E-GFP driven by *tubulin*-GAL4 is lethal.

### Analysis of CNS phenotypes in double and triple mutants lacking Ptp4E

To evaluate CNS defects in multiply mutant embryos, we stained them with a monoclonal antibody (mAb), 1D4, and also crossed a SemaIIB-τMyc reporter [29] into the mutant backgrounds. mAb 1D4, directed against the cytoplasmic domain of Fasciclin II [30], stains three longitudinal bundles on each side of the CNS in stage 17 embryos. Figure 3 shows 1D4 staining of the CNS in stage 17 wild-type, *Ptp4E*^1^, *Ptp10D*^1^, *Ptp4E*^1 ^*Ptp10D*^1^, and *Df(1)ovo4 Ptp10D*^1 ^embryos. In both of the *Ptp4E *double mutant genotypes, a mild fraying of the longitudinal bundles is observed. The outer bundle is often incompletely formed, and the outer bundle invades the middle longitudinal bundle. The phenotypes of the two genotypes are of approximately equal severity, suggesting that *Ptp4E*^1 ^could be a null mutation. Table 1 shows the quantitative comparison of the CNS phenotypes in double and triple mutant combinations.

**Table 1:** CNS axon phenotypes in *Rptp *double and triple mutant embryos

	Phenotype (%)			
	
Genotype	*n*	Two	One/none	Cross-over
*Ptp4E*^1^	128	2	0	0
*Ptp10D*^1^	128	1	0	0
*Ptp4E*^1 ^*Ptp10D*^1^; *UAS-4E-GFP/elav*	124	7*	0	0
*Ptp4E*^1 ^*Ptp10D*^1^	126	30^†^	0	1
*Ptp4E*^1^; *Ptp69D*^1^/*Df(3L)8ex25*	80	3	0	0
*Ptp10D*^1^; *Ptp69D*^1^/*Df(3L)8ex25*	128	63	21	100
*Ptp4E*^1 ^*Ptp10D*^1^; *Ptp69D*^1^/*Df(3L)8ex25*	106	54	16	100

CNS longitudinal axons were scored by counting the number of longitudinal tracts in each hemisegment at the midpoint between commissures (visualized by residual 1D4 staining at stage 17). In wild-type embryos, there are three fascicles per hemisegment. *n*, total number of hemisegments (T2–A6) scored at stage 17. Two: hemisegments with only two longitudinal tracts where the outer bundle is missing or is fused to the medial fascicle. One/none: hemisegments with only one or no longitudinal tracts. Cross-over: segments where axons abnormally cross the midline. *Expression of UAS-4E-GFP in neurons using the *elav*-GAL4 driver produces incomplete rescue (from 30% to 7%, versus 1–2% in single mutants). However, the statistical difference between *Ptp4E*^1 ^*Ptp10D*^1 ^and *Ptp4E*^1 ^*Ptp10D*^1^; *UAS-4E-GFP/elav *was highly significant (*p *< 0.0001, Chi-square test), indicating that pan-neural expression of Ptp4E rescues. ^† ^Statistical differences between *Ptp4E*^1 ^*Ptp10D*^1 ^and each of the single mutants were also significant (*p *< 0.0001, Chi-square test).

The CNS defects are specific to loss of Ptp4E, because they can be rescued by supplying wild-type Ptp4E in neurons. When we drove expression of the UAS-Ptp4E-GFP transgene with *elav*-GAL4, the break and fraying phenotypes were rescued to near-wild-type levels. The differences between *Ptp4E Ptp10D *and the rescued or wild-type embryos are both highly statistically significant (*p *< 0.0001; Table 1). There is also a subtle overexpression phenotype produced by driving Ptp4E-GFP in neurons, in which the longitudinal bundles have a 'wavy' appearance (data not shown).

SemaIIB-τMyc is a useful marker for a specific axon pathway that crosses the midline in the anterior commissure and then extends anteriorly within the longitudinal tract [29]. Staining with anti-Myc antibodies in embryos expressing this reporter in the *Ptp4E*^1 ^*Ptp10D*^1 ^background reveals that the longitudinal portions of the SemaIIB axon pathway exhibit a mild fraying, consistent with the 1D4 results (Figure 3).

Previous work from our group showed that *Ptp10D Ptp69D *mutants have a specific CNS phenotype in which some 1D4-positive longitudinal axons abnormally cross the midline. Two distinct longitudinal bundles usually remain in these mutants. The *Ptp10D Ptp69D *combination genetically interacts with *robo1*, *slit*, and *commissureless *mutations, and the data suggest that repulsive signalling through Robo1 in response to engagement of the midline Slit ligand is reduced in the absence of these RPTPs [3].

**Figure 4:**
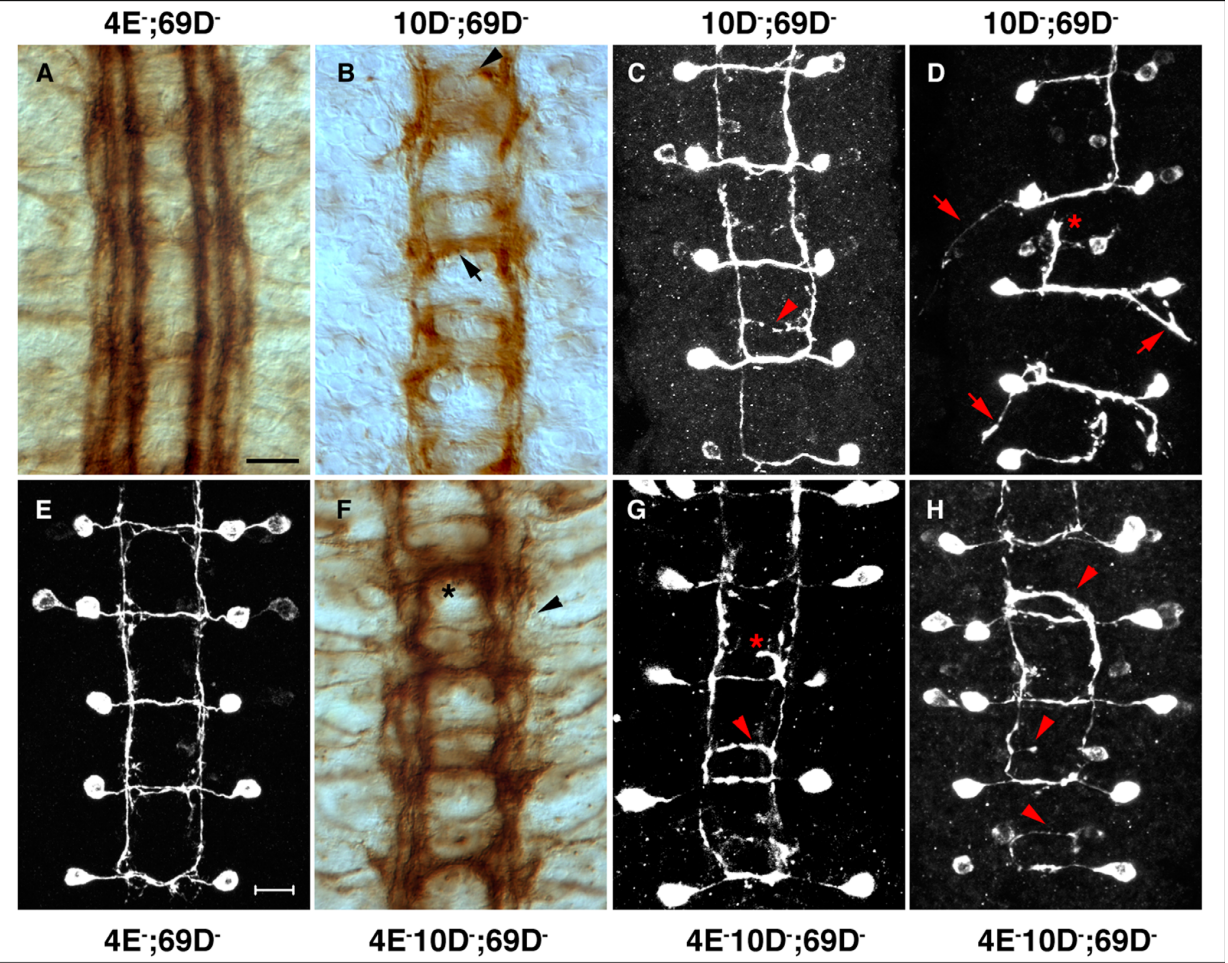
CNS axon guidance defects in double and triple mutants. Stage 17 embryos were stained with **(a,b,f) **1D4 and **(c-e,g,h) **anti-Myc as in Figure 3. (a) The *Ptp4E*, *Ptp69D *CNS shows very mild defects with 1D4 in which the outer longitudinal bundle is slightly wavy. (e) The SemaIIb-τmyc axons are almost indistinguishable from wild-type, although there is some slight fraying of the longitudinal segments of the pathways. (b) The *Ptp10D*; *Ptp69D *CNS has a strong ectopic midline crossing defect in which 1D4 axons grow across the posterior commissure (arrow); a thinner bundle is observed in the anterior commissure (arrowhead). Only one or two longitudinal bundles are visible, and the width of the CNS axon ladder indicates that the outer bundle is missing. (c) SemaIIb-τmyc axons in *Ptp10D*; *Ptp69D*. A thin axon bundle crosses the CNS in the posterior commissure (arrowhead). (d) An extreme *Ptp10D, Ptp69D *phenotype, with axons that project out of the CNS (arrows), and stalled growth cones along the longitudinal tract (asterisk). (f) 1D4 staining in *Ptp4E Ptp10D*; *Ptp69D *triple mutants show thick bundles that cross the midline in each segment (asterisk). There are two or three distinct longitudinal bundles (arrowhead). (g,h) Thick bundles of SemaIIb-τmyc axons in the triple mutant cross the midline in the posterior commissure (g, arrowhead; h, top arrowhead). A stalled axonal projection in this commissure is seen in (g, asterisk). Other abnormally crossing axons are indicated with arrowheads in (h). Anterior is up in all panels. Scale bars are 10 microns.

Since *Ptp4E *is similar to *Ptp10D*, we wondered whether *Ptp4E *might also genetically interact with *Ptp69D *to produce a synthetic CNS phenotype affecting the 1D4-positive longitudinal axons. This, however, is not the case. *Ptp4E Ptp69D *double mutants, like *Ptp69D *single mutants, have wild-type 1D4 and SemaIIB-τMyc patterns (Table 1, Figure 4a,e).

We then asked whether removal of both Ptp4E and Ptp10D together with Ptp69D would generate a new synthetic phenotype. We observed that the *Ptp4E Ptp10D; Ptp69D *triple mutant has a strong 1D4 phenotype, with extensive ectopic midline crossing (Figure 4f, Table 1). The 1D4 pattern shows midline crossing defects like those observed in *Ptp10D Ptp69D *mutants [3], in which a 1D4-positive bundle crosses in the posterior commissure, but the crossing bundle often is thicker than in the double mutants (Figure 4f, asterisk).

The longitudinal tracts look different from those in *Ptp10D Ptp69D *mutants, because they have more distinct bundles. In *Ptp10D Ptp69D*, two 1D4 bundles are observed that sometimes fuse into one, and the CNS is narrowed, suggesting that the outer bundle is missing (Figure 4b). In the triple mutants, there are always two, and sometimes three, longitudinal bundles. Extensive fusion and breakage of the bundles are observed, however (Figure 4f).

When examined with SemaIIB-τMyc, the *Ptp10D Ptp69D *double mutant displays occasional ectopic crossing by single axons in the posterior commissure (Figure 4c, arrowhead). Rare embryos have much more severe phenotypes in which the axons stall along the longitudinal pathways (Figure 4d, asterisk), and axons project out of the CNS rather than along the longitudinal tracts (Figure 4d, arrows). The triple mutant is observed to have more ectopic midline crossing in the posterior commissure than does the double mutant, and the crossing bundles are thicker (Figure 4g, arrowhead; Figure 4h). In one segment of Figure 4h, all of the axons that should project anteriorly appear to cross instead (top arrowhead). Other axons start crossing and then stall (Figure 4g, asterisk). We never observed SemaIIB axons that projected out of the CNS in triple mutant embryos (n = 10). It is difficult to say whether removing Ptp4E from a *Ptp10D Ptp69D *double mutant suppresses or enhances the double mutant phenotype. The phenotype changes, so that the outer 1D4 longitudinal bundle is restored in some segments of triple mutants. However, more ectopic crossing of SemaIIB axons is observed, and the ectopically crossing 1D4 bundles are often thicker.

What does this pattern of phenotypic interactions imply about the roles of these three RPTPs in CNS axon guidance? First, the existence of the synthetic *Ptp10D Ptp69D *phenotype indicates that Ptp10D and Ptp69D have redundant functions with regard to control of midline crossing, and that the activities of Ptp10D that are compensated for by Ptp69D with respect to midline crossing are not shared with Ptp4E. Second, there could be additional Ptp69D functions in CNS axonal guidance that are redundant with Ptp4E activities that are not shared between Ptp4E and Ptp10D. However, elimination of these activities in *Ptp4E Ptp69D *double mutants does not produce a strong 1D4 or SemaIIB phenotype. Third, when all functions of the Ptp10D/Ptp4E subfamily of RPTPs are eliminated, the 1D4 and SemaIIB phenotypes are still weak because these functions are mostly redundant with activities of Ptp69D. Finally, when all the Ptp10D/Ptp4E functions are removed together with the Ptp69D functions, the phenotype is subtly altered relative to the *Ptp10D Ptp69D *phenotype. The lack of a strong synthetic triple mutant phenotype suggests that Ptp4E is a relatively minor player in regulation of CNS axon guidance.

An earlier study from our group examined CNS axons in double, triple, and quadruple mutant combinations of *Lar*, *Ptp10D*, *Ptp69D*, and *Ptp99A *mutations [4]. Strikingly, in this paper it was found that *Lar Ptp69D Ptp99A *and *Ptp10D Lar Ptp99A *triple mutants both have an almost normal pattern of 1D4-positive longitudinal axons, but when the fourth RPTP is removed as well (in a *Ptp10D Lar Ptp69D Ptp99A *quadruple mutant) all of the 1D4-positive longitudinal pathways are converted to commissural pathways that cross the midline. Thus, Lar and Ptp99A can have a strong effect on CNS axon guidance, but only when they are removed together with both Ptp10D and Ptp69D.

We wondered whether analysis of other mutant combinations involving *Ptp4E *would produce results consistent with the idea that strong synthetic 1D4 CNS phenotypes are unique to combinations in which both *Ptp10D *and *Ptp69D *are mutant, as suggested by [4]. To examine this, we made *Ptp4E Lar, Ptp4E Ptp52F*, and *Ptp4E Ptp99A *double mutants, and *Ptp4E Ptp10D Lar *and *Ptp4E Ptp10D Ptp52F *triple mutants. *Ptp4E Lar *and *Ptp4E Ptp99A *mutants had no detectable alterations in 1D4-positive CNS longitudinal tracts. *Ptp4E Ptp10D Lar *mutants had phenotypes like those of *Ptp4E Ptp10D *double mutants, indicating that there are no important longitudinal axon guidance functions that are redundant between the Ptp10D/Ptp4E protein pair and Lar. *Ptp52 *mutants are the only *Rptp *single mutants that have a detectable 1D4 phenotype [5]. *Ptp4E Ptp52F *mutants had 1D4 phenotypes indistinguishable from *Ptp52F *single mutants, while *Ptp4E Ptp10D Ptp52F *triple mutants had stronger phenotypes. However, we had already demonstrated that *Ptp10D Ptp52F *double mutants have a more disorganized pattern of 1D4-positive bundles than *Ptp52F *single mutants [5]. The *Ptp4E Ptp10D Ptp52F *triple mutant does not show an obvious enhancement of phenotype relative to the *Ptp10D Ptp52F *double mutant (data not shown). In summary, of the eight mutant combinations involving *Ptp4E *that we examined, only the triple mutant that lacks both Ptp10D and Ptp69D has a strong CNS phenotype that is detectable with the markers used in this study. This phenotype is much like the *Ptp10D Ptp69D *double mutant phenotype, but differs from it in some subtle ways.

### Roles of the Ptp10D/Ptp4E subfamily in motor axon guidance

We also used mAb 1D4 staining to evaluate the motor axon phenotypes in all of these mutant combinations. There are approximately 36 motor neurons in each abdominal hemisegment. Motor axons exit the CNS in two main nerve roots (ISN and SN), then split into five distinct nerve branches. ISNd and SNc innervate ventral muscles, ISNb innervates ventrolateral muscles (VLMs), SNa innervates lateral muscles, and ISN innervates dorsal muscles. The patterns of motor axons in segments A2–A7 are essentially identical, so it is possible to quantitatively score phenotypes in up to 12 hemisegments per embryo.

Earlier work from our group and others showed that *Rptp *mutations affect every guidance decision made by these motor axon branches (with the exception of SNc branching, which has not been studied). *Lar *and *Ptp52F *are the only single mutants that have strong phenotypes. *Lar *mutations produce a 'parallel bypass' phenotype (approximately 30% penetrance in zygotic nulls), in which the ISNb axons leave the common ISN pathway at the exit junction but then fail to enter the VLM field. ISNd also fails to extend (approximately 80% penetrance) in these mutants [2,19]. *Ptp52F *regulates bifurcation of the SNa nerve. One of the two SNa branches (anterior/dorsal or posterior/lateral) is missing in approximately 40% of hemisegments in zygotic null mutants [5].

When double, triple, and quadruple combinations of *Rptp *mutations were analyzed, we observed that other decisions are perturbed in specific patterns. Each combination of mutations has a unique phenotype, and the data suggest that specific RPTPs are required for each pathway choice made by motor axon growth cones. This is described for ISNb guidance in the Background section. Another example is extension of the ISN past branchpoints in the dorsal muscle field. In *Lar *single mutants, the ISN usually reaches its normal termination point but arborization on muscle 1 is reduced. In the absence of both Lar and Ptp69D, the ISN stops at the second branchpoint near muscle 2, while in *Lar Ptp69D Ptp99A *triple mutants it stops at the first branchpoint near muscle 3 [19].

Not all combinations of *Rptp *mutations produce phenotypic enhancement. Ptp99A and Lar have opposing activities in controlling ISNb entry into the VLM field. Thus, in the strongest *Lar *mutant combination only 40% of ISNb branches successfully enter the VLM field, while in *Lar Ptp99A *double mutants this phenotype is almost completely suppressed, and approximately 96% of ISNbs enter the muscle field in a normal manner [4,19].

For the first four RPTPs to be analyzed (Lar, Ptp69D, Ptp99A, Ptp10D), it was possible to construct and analyze all 15 possible single, double, triple, and quadruple mutant combinations, and thus obtain a complete picture of the genetic relationships among these signalling molecules [4]. When we identified mutations in the fifth gene, *Ptp52F*, it became impossible to make and analyze every combination, so we examined only double mutants [5]. These two studies led to the conclusion that Ptp10D is relatively unimportant, relative to the other RPTPs, for regulation of motor axon guidance. This conclusion is opposite to that reached for CNS axon guidance, where we observe ectopic midline crossing only in genotypes where Ptp10D is missing [4].

Enhancement of *Ptp52F *phenotypes is observed in *Ptp10D Ptp52F *double mutants, so that ISNb 'stall' phenotypes, in which the ISNb stops short of its normal termination point at the ventral edge of muscle 12, and ISN truncation phenotypes are observed more frequently [5]. These are the only double mutants involving *Ptp10D *that exhibit phenotypic enhancement relative to the corresponding single mutant. *Ptp10D *mutations increase the penetrance of some double and triple mutant motor axon phenotypes involving *Lar*, *Ptp69D*, and *Ptp99A*, and decrease the penetrance of others [4].

We wondered whether this apparent lack of a role for Ptp10D in motor axon guidance arises from the fact that Ptp10D is the only RPTP in *Drosophila *that has a paralog within the same subfamily. Each of the other RPTPs appears to represent its own subfamily. The Lar subfamily has a representative in *C. elegan*s and three members in mammals, while Ptp69D and Ptp99A each have one worm counterpart but no obvious mammalian orthologs [5]. Ptp52F has similarities to type III RPTPs, but has a unique amino-terminal sequence and appears to be unique to drosophilids (unpublished results). Because of their close sequence relationship, the loss of Ptp10D function in the motor axon system might be compensated for by the presence of Ptp4E. This model predicts that *Ptp4E *double mutant combinations also would not exhibit phenotypic enhancement relative to the corresponding single mutants. Indeed, we found this to be the case: no motor axon phenotypes are observed in *Ptp4E Ptp99A *mutants, and the phenotypes seen in *Ptp4E Ptp69D*, *Ptp4E Lar*, and *Ptp4E Ptp52F *mutants are no stronger than in the corresponding *Ptp69D*, *Lar*, and *Ptp52F *single mutants. We also found that removal of both members of the Ptp10D subfamily, in *Ptp4E Ptp10D *double mutants, does not produce motor axon phenotypes, suggesting that the subfamily has no essential role in motor axon guidance that is not compensated for by the presence of one of the other RPTPs (Table 2 and data not shown).

**Table 2:** Motor axon phenotypes in *Rptp *double and triple mutant embryos

	Phenotype (%)*					
	
Genotype						
**ISN**	*n*	*t*	T	SB	FB	
*Ptp4E*^1 ^*Ptp10D*^1^	155	6	3	3	0	
*Ptp4E*^1^*; Ptp52F*^18.3^	126	12	2	8	2	
*Ptp10D*^1^*; Ptp52F*^18.3^	207^†^	67	40	23	4	
*Ptp4E*^1 ^*Ptp10D*^1^*; Ptp52F*^18.3^	203	74	11	60^‡^	3	
*Dlar*^5.5^*/Dlar*^13.2^	256^§^	41	22	19	0	
*Ptp4E*^1^*; Dlar*^5.5^*/Dlar*^13.2^	91	51	30	21	0	
*Ptp4E*^1 ^*Ptp10D*^1^*; Dlar*^5.5^*/Dlar*^13.2^	133	60	32	26	2	
						
**ISNb**	*n*	t	B	S	C	
*Ptp4E*^1 ^*Ptp10D*^1^	119	2	0	2	0	
*Ptp4E*^1^*; Ptp69D*^1^*/Df(3L)8ex25*	106	11	8	3	0	
*Ptp10D*^1^*; Ptp69D*^1^*/Df(3L)8ex25*	101	94	4	90^¶^	0	
*Ptp4E*^1 ^*Ptp10D*^1^*; Ptp69D*^1^*/Df(3L)8ex25*	108	93	1	36^¶^	56^¶^	
						
**SNa**	*n*	t	M	S	A	-
*Ptp4E*^1 ^*Ptp10D*^1^	139	5	3	1	1	0
*Ptp4E*^1^*; Ptp69D*^1^*/Df(3L)8ex25*	122	7	5	1	1	0
*Ptp10D*^1^*; Ptp69D*^1^*/Df(3L)8ex25*	85	26	13	10	2	1
*Ptp4E*^1 ^*Ptp10D*^1^*; Ptp69D*^1^*/Df(3L)8ex25*	106	41	18	14	0	9^¥^

*n*, number of hemisegments (A2–A7) scored; *t*, all affected branches. For ISN phenotype categories: T, ISN branches that end at the normal terminal arbor position but are thin or bifurcated; SB, ISNs that terminate at the second branchpoint; FB, ISNs that terminate at the first branchpoint. For ISNb phenotype categories: B, ISNb branches that bypass the ventrolateral muscle field (muscles 6, 7, 12, 13) and grow adjacent to ISN or fail to exit the ISN pathway and are fused to ISN; S, ISNbs that stall within the VLM field or fail to enter the VLM field; C, ISNbs that stall with a clump. For SNa phenotype categories: M, either posterior or anterior branch is missing; S, SNa branches stall near the bifurcation point; A, additional branches;-, the whole branch is very thin or absent. *The numbers listed are percent of hemisegments that display a phenotype. ^†^The results for *Ptp10D*^1^; *Ptp52F*^18.3 ^were published in [5]. ^‡^For ISN motor axons, the differences between the triple mutant and each of the double mutants (*Ptp4E Ptp52F *and *Ptp10D Ptp52F*) are statistically significant (*p *< 0.0001, Chi-square test). ^§^The results for *Dlar*^5.5^*/Dlar*^13.2 ^were published in [19]. ^¶^While approximately 90% of ISNbs end at the base of m13 in both genotypes, the phenotype is different in the triple mutant: 56% of stalled ISNb axons in triple mutants form a clump, whereas in the double mutant they simply resemble earlier (stage 16) axons with growth-cone like morphologies. These embryos were scored with genotypes blinded by another observer to make sure we could distinguish the phenotypes. The difference between the triple and the double mutants is highly statistically significant (*p *< 0.0001, Chi-square test). ^¥^Difference between the triple and the double mutant (*Ptp10D Ptp69D*) is statistically significant (*p *< 0.0001, Chi-square test).

We then asked whether strong phenotypic interactions might be observed in triple mutants lacking both Ptp4E and Ptp10D together with one of the other subfamilies. If so, this would indicate that there are motor axon guidance functions that are redundant between the Ptp10D subfamily and another subfamily, so that synthetic phenotypes would be observed only when both subfamilies are eliminated. To examine this question, we analyzed the triple mutants described above for motor axon phenotypes. Our data show that *Ptp4E Ptp10D Lar *mutants do not have any new phenotypes, and also do not exhibit phenotypic enhancement relative to *Lar *single mutants (Table 2). Thus, as for CNS axon guidance, there appear to be no important functions of the Ptp10D/Ptp4E subfamily that are uncovered by removal of Lar.

**Figure 5:**
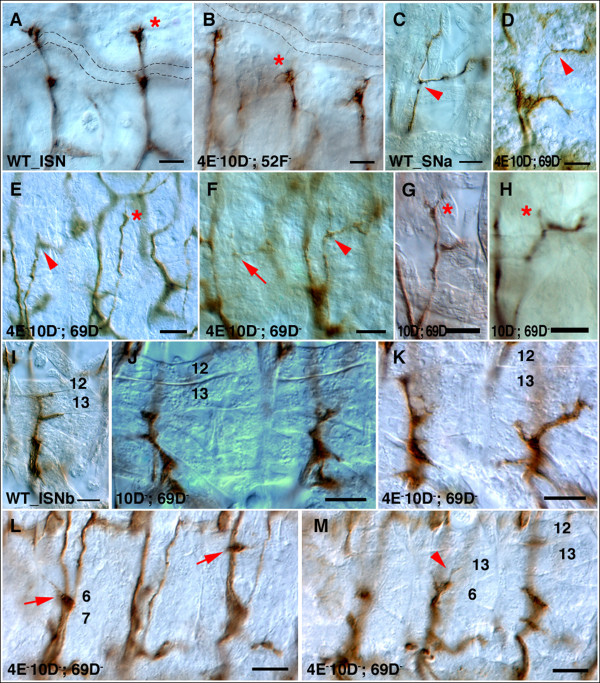
Motor axon defects in triple mutants. Stage 16 or 17 embryos were stained with 1D4 and visualized using HRP immunohistochemistry. **(a) **In wild-type stage 16 embryos, ISN reaches its target and synapses with muscles 1 and 9 (asterisk) at a position distal to the tracheal dorsal trunk (shown in dotted lines). **(b) **In a *Ptp4E Ptp10D*; *Ptp52F *triple mutant embryo, ISN stalls at the second branch point (asterisk). **(c) **Wild-type SNa bifurcates (bifurcation point shown in arrowhead) and extends an anterior branch (left) and a posterior branch (right). **(d-f) **In *Ptp4E Ptp10D*; *Ptp69D *triple mutants, SNa shows various defects. Some branches are very thin (d, arrowhead) or almost undetectable (f, arrow). The branch on the right in (f, arrowhead) is normal. The left SNa in (e) has only stubs at the branchpoint (arrowhead). The SNa on the right abnormally projects beyond the muscle 22/23 bifurcation cleft and sends a thin projection toward an abnormal ISN side branch (e, asterisk). **(g,h) **The SNa in *Ptp10D*; *Ptp69D *embryos. Extra branch is shown on the anterior branch (g, asterisk), and a very short anterior branch is observed in (h, asterisk). **(i) **Wild type ISNb. At stage 17, synapses have begun to form at the muscle 12/13 cleft and at the bottom of muscle 13. **(j) ***Ptp10D*; *Ptp69D *stage 17 embryo shows ISNb branches with growth-cone-like morphologies that end at muscle 13. **(k-m) **ISNb defects in *Ptp4E Ptp10D*; *Ptp69D *triple mutants. (k) The ISNb on the right has an abnormal trajectory and stalls at muscle 13. (l,m) The same hemisegments, imaged in different focal planes. The characteristic clumped phenotype is seen in the left hemisegment (l, left arrow). The right hemisegment has a stall/bypass phenotype in which some ISNb axons grow out along the ISN, producing an ectopic branch onto muscle 12 (l, right arrow). In the middle hemisegment, thin branches also emerge from the clump and grow onto the surface of muscle 13 (m, arrowhead). Anterior is left and ventral is down in all panels. All scale bars are 10 microns.

For the other two triple mutant combinations, however, we did observe phenotypic enhancement by removal of both Ptp4E and Ptp10D. *Ptp4E Ptp10D Ptp69D *triple mutants have a stronger ISNb phenotype than any of the component double mutants. This is a 'clump' phenotype in which 90% of ISNbs terminate in a darkly staining blob at the dorsal border of muscle 6 (Figures 5l,m). In cases where the ISNb passes this point, it is often misrouted (Figure 5k, right hemisegment), or bypasses the muscle field (Figure 5l) by growing along the ISN. As in bypass phenotypes seen in other mutants, the axons often ectopically project interiorly to muscle 12 from the ISN (Figure 5l, right arrow), suggesting that they retain an affinity for this muscle. *Ptp10D Ptp69D *double mutants have a similar percentage of ISNb branches that fail to reach muscle 12 (stall phenotype); however, these branches maintain a growth cone-like appearance (Figure 5j).

In almost every affected triple mutant hemisegment, the ISNb clump is at the same point. These data suggest that extension of ISNb axons onto muscle 13 might require a specific signalling event(s) that can be mediated by either a type III RPTP or by Ptp69D. In contrast, for most other published *Rptp *stall phenotypes (for example, *Ptp69D Ptp99A *[1,19]) defective ISNbs terminate at a variety of sites, suggesting that loss of the RPTPs produces multiple defects in pathway choice and extension that occur in a probabilistic manner. The differences between the triple and double mutant phenotypes are highly statistically significant (*p *< 0.0001; Table 2).

We also observed a subtle phenotypic enhancement for SNa defects. We previously showed that *Ptp10D Ptp69D *double mutants have a SNa defect in which one of the branches is missing (13% penetrance of a completely absent branch). For a smaller percentage of SNas (10%), both branches are missing or the SNa fails to even reach the bifurcation point. In *Ptp4E Ptp10D Ptp69D *triple mutants, the penetrances of the missing branch phenotypes are similar to those in the double mutant, but an additional 9% of SNa nerves are very thin (Figure 5f, arrow) or completely absent; this phenotype is not seen in *Ptp10D Ptp69D *(*p *< 0.0001; Table 2) Also, there are SNa nerves that project along abnormal pathways (Figure 5d).

The other major effect on motor axon guidance we observed in response to loss of Ptp4E was enhancement of a *Ptp10D Ptp52F *phenotype in which the ISN is truncated at the second branchpoint. This phenotype is observed in about 20% of hemisegments in the double mutant. However, the penetrance of second branchpoint truncation increases to 60% in the *Ptp4E Ptp10D Ptp52F *triple mutant (*p *< 0.0001; Figure 5b, Table 2). For the other phenotypes of *Ptp10D Ptp52F *(SNa bifurcation failure and ISNb stalling), we saw only small increases in phenotypic penetrance when Ptp4E is removed.

## Conclusion

In *Drosophila*, five of the six RPTPs were reported to be neural-specific in late embryos, and all the zygotic *Rptp *phenotypes that have been published are axon guidance alterations. In contrast, many of the 17 mammalian RPTPs are expressed in non-neural cell types and have a variety of functions unrelated to axon guidance. Since *Ptp4E *is the only widely expressed *Rptp *gene, we speculated that studying its mutant phenotype might reveal new functions for *Drosophila *RPTPs outside the nervous system, and that these might provide information about functions of mammalian non-neural RPTPs. One might have expected that *Ptp4E *mutations would cause lethality and produce strong phenotypes, since no other RPTPs would be able to compensate for the loss of Ptp4E in non-neural cells. This, however, is not the case. *Ptp4E *mutants are viable, fertile, and apparently healthy, and have no detectable phenotypes in the nervous system or elsewhere. Furthermore, our evolutionary analysis indicates that *Ptp4E *is a relatively recent invention; it is present in drosophilids but not in mosquitoes or non-dipteran arthropods. Within the drosophilids, its sequence also changes more rapidly than that of *Ptp10D*, suggesting that it has been less constrained by evolution (Figure 1). All of these considerations indicate that Ptp4E is not essential for development of non-neural cell types in *Drosophila*.

Perhaps in *Drosophila *the functions executed by mammalian RPTPs in non-neural cell types are carried out by one or more of the eight nonreceptor PTPs. Some of these are ubiquitously expressed. Only three have been genetically characterized. Csw and PTP-ER are involved in cell fate determination [31,32]. Mutations in *ptpmeg *produce axonal defects in the adult brain [33]. Ptpmeg, however, does not act in the neurons that exhibit the axonal phenotypes, but is required in surrounding cells [33]. Thus, it is unlikely to participate in growth cone signal transduction in the same manner as the RPTPs.

Ptp10D and Ptp4E are the only *Drosophila *RPTPs that are members of the same subfamily; the other four are each the sole fly representative of their subfamily. Mutations in three of the other four *Rptp *genes (*Lar*, *Ptp52F*, *Ptp69D*) cause lethality. This suggests that the viability of *Ptp10D *and *Ptp4E *single mutants might be due to compensation by the other member of the subfamily, and that a *Ptp4E Ptp10D *double mutation would cause lethality. This is in fact observed; the double mutant dies at hatching. However, it does not have generalized defects. Rather, the defects we have found are all within the nervous system and the tracheal network. Our unpublished data show that Ptp10D is also selectively expressed in tracheal cells (MJ and KZ, manuscript in preparation). We suggest that *Ptp4E Ptp10D *double mutant phenotypes are observed only where Ptp10D is expressed.

**Figure 6:**
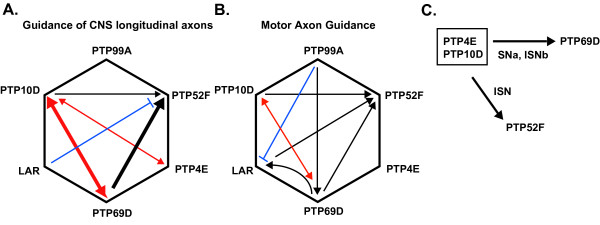
Summary of pairwise genetic interaction among all six RPTPs. Matrices depict role of RPTPs in CNS longitudinal axon guidance **(a) **and motor axon guidance **(b)**. **(c) **Interaction of *Ptp4E Ptp10D *double mutant with RPTPs. *Ptp4E *shows enhancement of motor axon guidance phenotypes observed in *Ptp10D Ptp69D *and *Ptp10D Ptp52F *double mutants. Thicker lines indicate stronger genetic interaction than thinner lines. Red double headed arrows indicate synthetic genetic interactions. Black single headed arrows indicate enhancement/cooperation, and the blue lines indicate genetic suppression.

The analysis described in this paper, together with that in several other papers from our group [4,5,19] allow us to assemble complete genetic interaction matrices for pairwise combinations of mutations in all six of the *Rptp *genes. Figure 6a is a matrix depicting the functions of the RPTPs in regulation of longitudinal axon guidance in the CNS, as assayed by 1D4 staining. The lines represent different types of genetic interactions. Red double-headed arrows indicate synthetic phenotypes, where neither of the single mutants exhibits a detectable phenotype but the double mutant has a phenotype; the arrow thickness indicates the strength of the phenotype. These are seen for *Ptp10D Ptp69D *[3] and *Ptp4E Ptp10D *(Figure 3). Black arrows indicate enhancement of a single mutant phenotype by removal of a second RPTP. In the CNS, these are observed only for *Ptp52F*, since this is the only single mutant that has a CNS phenotype detectable with 1D4. Finally, blue lines with bars at the end indicate suppression, where removal of a second RPTP suppresses the single mutant phenotype. This is observed for *Lar Ptp52F *mutants [5], and may indicate that these two RPTPs function in a competitive manner (in a formal genetic sense) to regulate a CNS signalling pathway.

Figure 6b shows the interaction matrix for motor axon guidance. This is different from the CNS interaction matrix, so we can conclude that the relationships between the RPTP signalling pathways differ in some cases between motor neurons and CNS interneurons. However, *Ptp10D Ptp69D *double mutants have a synthetic SNa phenotype [4], so these two RPTPs interact strongly in regulating both CNS and motor axon guidance. Loss of Ptp10D also enhances both the CNS and motor axon defects of *Ptp52F *mutants [5].

As in the CNS, there are competitive relationships between RPTPs, but they are seen for a different RPTP pair. In motor axons, removal of Ptp99A completely suppresses the *Lar *ISNb parallel bypass phenotype [19]. *Lar *mutations enhance the *Ptp52F *motor axon phenotypes rather than suppressing them as they do in the CNS [5].

In this paper, we have defined the phenotypes associated with simultaneous elimination of the functions of two RPTP subfamilies, by examining triple mutants removing both Ptp4E and Ptp10D together with each of the other three RPTPs whose absence produces lethality. This analysis shows that the Ptp10D/Ptp4E subfamily is redundant with Ptp69D in controlling guidance decisions made by three neuronal types, but *Ptp4E *mutants have relatively minor effects relative to *Ptp10D *mutants. For guidance of 1D4 and SemaIIB axons within the CNS, removal of both members of the Ptp10D/Ptp4E subfamily together with Ptp69D modulates the phenotype observed in *Ptp10D Ptp69D *mutants (Figure 4). For SNa axons, the triple mutant has an enhanced phenotype, in that 10% of SNa nerves now fail to extend altogether; this is almost never observed in double mutants (Figures 5 and 6c). We also observe enhancement of a *Ptp10D Ptp52F *ISN truncation phenotype by removal of Ptp4E (Figures 5 and 6c), but no strong interactions between *Ptp4E Ptp10D *and *Lar *are observed in the CNS or neuromuscular system.

These results suggest that there is a special relationship between the Ptp10D/Ptp4E subfamily and Ptp69D. Perhaps these two types of RPTPs have similar substrates in both CNS interneurons and motor neurons. In CNS neurons, some critical substrate(s) dephosphorylated by Ptp69D might also be dephosphorylated by either Ptp10D or Ptp4E, so that certain phenotypes, such as crossing of all the SemaIIB axons in the wrong commissure (Figure 4h), are observed only when all three RPTPs are eliminated. However, in CNS neurons such as the neuroblast 2–5 lineage, whose axons ectopically cross the midline in *Ptp10D Ptp69D *double mutants [3], Ptp4E cannot compensate for the loss of Ptp10D. Perhaps in these cells the relevant Ptp69D substrate(s) can be dephosphorylated by Ptp10D but not by Ptp4E; however, this seems unlikely given that Ptp4E and Ptp10D have PTP domains that are much more similar to each other than are those of Ptp69D and Ptp10D. Alternatively, perhaps the Ptp4E concentration is too low in these neurons for efficient dephosphorylation to occur. Another possibility is that growth cones of these neurons contact Ptp10D ligands, but not Ptp4E ligands, and that ligand contact is required for signalling. An understanding of the biochemical origins of these genetic interactions will require identification and characterization of RPTP ligands, substrates and downstream signalling proteins, as well as localization of these proteins to specific neuronal types.

## Materials and methods

### *Drosophila *stocks and transgenic flies

*Ptp4E*^1 ^was generated by imprecise excision of EP425 [34] inserted 1,157 nucleotides upstream of the transcription start. PCR was used to map the deletion endpoints. The deletion removes approximately 10.5 kb, starting from 130 bp upstream of the EP425 insertion site to before the start of exon 3. The excision event introduced chromosomal rearrangement, and as a result there are unrecognizable sequences between the endpoints.

The following *Rptp *mutant flies were used: *Ptp10D*^1 ^[3]; *Ptp69D*^1 ^and *Df(3L)8ex25 *[1]; *La*^*r*5.5 ^*and Lar*^13.2 ^[2]; *Ptp52F*^18.3 ^[5]. Lethal mutations were balanced over GFP balancer chromosomes for sorting homozygous mutant embryos. Double and triple mutant lines were checked for the presence of *Ptp4E*^1 ^by PCR. *Elav*-GAL4 was obtained from Bloomington Stock Center (Bloomington, IN, USA). All crosses were carried out at 25°C.

The UAS-Ptp4E-GFP construct was made by PCR amplification of the entire coding region of the long Ptp4E cDNA, Ptp4E-A, and cloning it into the Gateway System vector (DGRC, Bloomington, IN, USA). The vector used placed a GFP sequence downstream of the Ptp4E coding sequence. The entire construct was sequenced, and also expressed in S2 cells to check for GFP expression to verify that PCR errors were not introduced into the clone. Ptp4E-GFP DNA was injected into embryos using standard methods to generate transgenic flies. Multiple lines were generated and examined; UAS-4E-GFP#4/CyO was used for all studies.

### Generation of Ptp4E antibodies

To generate antibodies against the native protein, we made a construct 4E-AP that was expressed in insect cells using a baculovirus expression system. The 4E-AP construct was made by dropping in a 2.7 Psi I/Kpn I fragment from Ptp4E-A (extracellular domain amino acids 169–1073) into a modified baculovirus expression vector pAcGP67-A (BD Biosciences, San Jose, CA, USA). The vector contains a pg67 secretion signal sequence followed by a 6x-His tag, placed upstream of the Ptp4E insertion site. The sequence for human placental alkaline phosphatase was placed in frame, carboxy-terminal to the Ptp4E sequence. The protein expressed consists of the gp67 signal peptide-6xHis-Ptp4E-AP fusion protein that was harvested from insect cell media. Protein was concentrated ten-fold, then purified over a Ni-NTA column (Qiagen, Valencia, CA, USA). Conditions used are: binding buffer (10 mM imidazole, 300 mM NaCl, 20 mM, Tris pH8.0), wash buffer (25 mM imidazole, 300 mM NaCl, 20 mM, Tris pH8.0), and elution buffer (250 mM imidazole, 300 mM NaCl, 20 mM, Tris pH8.0). Purified protein was injected at approximately 100 μg per boost. Antibodies were generated at the Caltech Monoclonal Facility.

### Whole-mount *in situ *hybridization and immunohistochemistry

Embryos were collected overnight, fixed and hybridized with digoxigenin-UTP antisense RNA probe. Probes were generated from approximately 750 bp PCR product amplified from *Ptp4E *cDNA that corresponds to the first four exons. Conditions were used as previously described in [35] with some modifications.

Whole-mount antibody staining of stage 16–17 embryos was performed using standard procedures [36], with some variations. Embryos were fixed for 20 minutes with 4% paraformaldehyde. Washes were done in 0.1% PT (0.1% Triton X-100 in phosphate-buffered saline), and blocking buffer used was PT with 0.1% bovine serum albumin + 5% normal goat serum. Double and triple mutant embryos were balanced over GFP balancer chromosomes and homozygous mutants were sorted for lack of GFP expression. Anti-GFP and mAb 1D4 were simultaneously incubated on embryos. Alexa-488 conjugated secondary antibodies were used against anti-GFP, and non-green embryos were sorted under an epifluorescent dissecting microscope. These embryos were then treated with horseradish peroxidase (HRP) immunohistochemistry using standard procedures [36] to visualize 1D4 staining pattern.

In cases where RPTP specific antibodies were used for sorting, staining was done in a sequential manner. Embryos were first incubated with RPTP antibodies, sorted, and then incubated with 1D4. For some of the triple mutant combinations, live embryos were sorted under the epifluorescent dissecting microscope, then dissected live on Superfrost/Plus adhesion slides (Fisher Scientific, Pittsburgh, PA, USA) in phosphate-buffered saline. These live dissected embryos were fixed on the slide, and carried through the same procedures as whole-mount embryo staining procedure.

Primary antibodies were used at the following dilutions: anti-GFP (Invitrogen, Carlsbad, CA, USA) at 1:1,000, mAb 1D4 at 1:3, mouse anti-Ptp4E (polyclonal) at 1:500, mouse anti-Ptp10D (8B2) at 1:3; mouse anti-Ptp69D (2C2) at 1:3, mouse anti-Myc (clone 9E10, Sigma, St. Louis, MO, USA) at 1:250. Secondary antibodies used were: Alexa Fluor 488 and Alexa Fluor 568 (Invitrogen) at 1:1,000, HRP-conjugated goat anti-mouse IgG+IgM (Jackson ImmunoResearch Laboratories, Inc., West Grove, PA, USA). Samples were mounted on Vectashield (Vector Laboratories, Burlingame, CA, USA) or glycerol. Samples were photographed on the Zeiss Axioplan microscope using DIC optics. Fluorescent images were taken on Zeiss LSM510, and images were processed using Adobe Photoshop.

### Phylogenetic tree

Phylogenetic tree construction was performed using the software Geneious Pro, available at [37]. The accession numbers of sequences are the following. Ptp4E-PA: AAF45998 (*melanogaster*), CH940655 (*virilis*), CH902621 (*ananassae*). Ptp10D-PA: AAS65319 (*melanogaster*), CH940655 (*virilis*), CH902630 (*ananassae*). *Aedes aegypti*, AAEL012083. *Anopheles gambiae*, AGAP004246. *Apis mellifera*, GB19351.

## Competing interests

The author(s) declare that they have no competing interests.

## Authors' contributions

MJ carried out the genetic and molecular studies, and wrote the paper together with KZ. HN participated in the genetic studies and sequence alignment/evolutionary studies. SB isolated the *Ptp4E *mutant allele and isolated the full-length cDNA. KZ participated in the genetic studies and wrote the paper together with MJ. All authors read and approved the final manuscript.
